# How to Improve the Cooperation Mechanism of Emergency Rescue and Optimize the Cooperation Strategy in China: A Tripartite Evolutionary Game Model

**DOI:** 10.3390/ijerph19031326

**Published:** 2022-01-25

**Authors:** Jida Liu, Yuwei Song, Shi An, Changqi Dong

**Affiliations:** School of Management, Harbin Institute of Technology, Harbin 150001, China; kittadada@yeah.net (J.L.); 21B310020@stu.hit.edu.cn (Y.S.)

**Keywords:** emergency rescue, emergency cooperation, evolutionary game theory, emergency management, emergency capital stock

## Abstract

To reveal the interaction and influence mechanism between emergency rescue entities, and to explore and optimize a cooperation mechanism of emergency rescue entities, a tripartite evolutionary game model of emergency rescue cooperation based on government rescue teams, social emergency organizations, and government support institutions was constructed. The stability of each game subject’s strategy choice was explored. Simulation analysis was applied to investigate the influence mechanism of key parameters on the evolution of the game subject’s strategy combination. The research results show that government rescue teams, social emergency organizations, and government support institutions have consistent political demands and rescue targets in emergency rescue cooperation. The game subjects are driving forces for each other to choose positive strategies. The game evolution process of the emergency cooperation model shows a “mobilization-coordination” feature. At the same time, the emergency capital stock formed based on trust relationships, information matching, and institutional norms between game subjects can promote the evolution of the game system toward (1,1,1). In addition, for government organizations with limited emergency resources, the average allocation of emergency resources is not the optimal solution for emergency rescue efficiency. However, it is easier to achieve the overall target of emergency rescue cooperation by investing limited emergency resources in key variables that match the on-site situation. On this basis, combined with the practice of emergency rescues in emergencies, countermeasures and solutions are proposed to optimize the mechanism and improve the efficiency of emergency rescue cooperation.

## 1. Introduction

In recent years, with the superposition and coupling of various risk factors, emergencies have shown more obvious complex characteristics, such as intersections, derivatives, chains, and randomness. It has been difficult for the previous management mode of relying on a single government department to effectively manage all kinds of emergencies [1,2]. Strengthening cooperation and coordination among emergency organizations is an effective way to manage emergencies. In the practice of emergency management, the Chinese government has gradually formed institutional and voluntary emergency cooperation mechanisms [3]. Meanwhile, further exploring cross-organization, the cross-sector mode, and its characteristics formed between emergency organizations has become an important topic in the field of emergency management research [4,5,6]. As an important component of emergency management, an emergency rescue is the most direct link to protect the safety of people’s lives and property, reduce the losses of emergencies, and prevent the occurrence of secondary disasters, which are remarkable characteristics of an emergency cooperation mechanism.

At present, there are still outstanding problems in China’s emergency rescue system, such as the imperfect cooperation mechanism between emergency rescue subjects, the mismatch between support strategies and rescue operations, and the “disorderly” participation of social emergency organizations. The practice of emergency rescues shows that it is an important basis to improve the overall efficiency of government emergency management to clarify the key tasks and strategies of emergency rescue subjects, improve the cooperative relationship between emergency rescue subjects, and strengthen the planning and adaptive evolution mechanism of emergency rescues [7,8]. However, existing studies mostly focus on the formation and characteristics of the government’s emergency cooperation mode in an emergency response, and there are few dynamic evaluations and targeted studies of cooperation strategies from the perspective of emergency rescues. At the present stage, the framework of China’s emergency rescue force system has taken initial shape, and the coordination mechanism between emergency management departments and the emergency function support departments of communication functions, grain storage functions, electric power functions, and energy functions has been further improved. Meanwhile, after the exploration and development of the Wenchuan earthquake, the COVID-19 epidemic, and the explosion accident in Tianjin Binhai New Area, the enthusiasm of social emergency organizations to participate in emergency response has continued to rise. All these facts provide timely and appropriate research opportunities for us to explore and optimize the cooperation mechanism of emergency rescue subjects, especially to reveal the interaction and influence mechanism of strategy selection among emergency rescue subjects.

Therefore, the key problems to be solved in this article are to clarify the main types of emergency rescue subjects, identify the cooperation mechanism between these emergency rescue subjects, and analyze the restricting factors that affect the cooperation relationship between them. Meanwhile, through the practice of emergency rescues, this article will propose countermeasures and solutions to break the bottleneck of emergency rescue organization cooperation, enrich the cooperation mode of emergency rescues, optimize the cooperation mechanism of emergency rescues, and improve the cooperation level of emergency rescues. It is hoped that the suggestions can enhance the supporting role of social forces in the government’s emergency management and strengthen the overall level of the government’s response to emergencies, to reduce the losses and negative impacts of emergencies on social security and economic development.

At the beginning of the discussion of the cooperation mechanism, we summarize the past emergency rescue practices and divide the emergency rescue subjects into the government rescue team, the government support institution, and the social emergency organization in emergency rescue, which are the core objects of our research. The goal of this article is to explore the interaction and evolution trend of the three types of subjects in the process of emergency rescue cooperation. By studying the cooperation mechanism, a feasible strategy for coordinating the three types of subjects will be proposed.

Game theory is usually used to describe the cooperative relationship between organizations, especially the choice of organizational strategies from the perspectives of rewards and punishments, reciprocity, etc. Adopting the evolutionary game method is instructive for explaining the dynamic process of strategy selection among the three subjects in the process of emergency rescue cooperation. Therefore, this article will try to use the evolutionary game method to study the interaction between different subjects in emergency rescue cooperation and its evolution process.

The other parts of this article are arranged as follows: Section 2 summarizes the research on emergency rescue cooperation and the applicability of evolutionary game theory in the study of organizational relations in emergency responses. Section 3 puts forward and explains the sources of the research questions, the hypotheses and parameters of the emergency rescue cooperation game model, and the replicative dynamic system of the game model. Section 4 discusses the strategic stability of the government rescue team, the social emergency organization, and the government support institution in the cooperative game of emergency rescues. In Section 5, the influence mechanism of parameters on the evolution of the game system is analyzed by numerical simulation. Section 6 discusses the applicability of the emergency rescue cooperation game model and the research results. Section 7 summarizes the research conclusions and puts forward suggestions to improve the efficiency of emergency response.

## 2. Literature Review

How emergencies can be effectively managed has always been an important research question in the field of emergency management. In recent years, inter-organizational cooperation in emergency management has been widely considered by the academic community and has experienced rapid development [9]. Existing studies have also fully confirmed the key role of emergency cooperation mechanisms in managing emergencies [10]. Inter-organizational cooperation in emergency responses can facilitate the establishment of effective forms of coordination and communication between different types of organizations. It can also realize resource sharing and cost savings in emergency management. Practice and theory have verified that emergency cooperation and organizational coordination run through the whole process of emergency management [11,12], including prevention and emergency preparedness, monitoring and early warnings, emergency disposal and rescues, and post-event recovery and reconstruction. In the field of emergency cooperation, existing studies have carried out in-depth discussions on safety supervision cooperation, early warning information release cooperation, emergency response cooperation, emergency disposal cooperation, and other areas. The characteristics of emergency cooperation in response to different types of emergencies are also explored based on actual cases. The practical cooperation mechanism construction modes of emergency management are then summarized and put forward. From the perspective of an emergency cooperation mechanism, the main research direction is to optimize the emergency cooperation mechanism at the same level and within the region and to construct a new emergency cooperation mechanism across the region and at different levels. Emergency cooperation between the government and social organizations, cross-regional emergency cooperation, and cross-military-civilian emergency cooperation are novel practices in the study of emergency cooperation mechanisms [13,14,15].

An emergency rescue is a mandatory administrative emergency measure. Carrying out emergency rescue operations is one of the most important functions of the government in emergency management. Generally, emergency rescue operations are mainly composed of rescue measures, control measures, support measures, preventive measures, mobilization measures, and stability measures. The implementation of emergency rescues often requires on-site commands, search-and-rescue personnel, on-site protection, material support, medical treatment, and other work. It is difficult for a single government organization to manage emergencies and complete emergency rescue operations alone [16,17].

Therefore, participants of emergency rescues have carried out multi-dimensional and multi-level rescue cooperation through information communication, resource integration, task allocation, and other modes [18,19]. The difficulty and complexity of emergency rescues are determined by the unpredictability, uncertainty, non-routine, and extensive influence of emergencies. At present, the existing studies often focus on actual cases and emergency rescue practice, and they systematically comb and summarize the current problems and practical experience of emergency rescue force allocation [20], emergency rescue facilities locations [21], emergency rescue logistics optimization [22,23], emergency rescue function matching [24], emergency rescue capability assessment [25], and the unified allocation of emergency rescue resources [26]. The main aims of the research on emergency rescue cooperation are to explore the establishment of a comprehensive emergency rescue force system and to design a coordination mechanism with organizational characteristics and rescue practices. In terms of the types of emergency rescue organizations, in addition to the government rescue teams, social emergency organizations and the government support institutions are also important components of emergency rescue operations.

On the one hand, the social emergency organization is an indispensable part of China’s emergency management system [27]. The orderly degree of participation and execution of the social emergency organization in emergency rescue operations have become important factors in measuring the effectiveness and level of governments’ management of emergencies [28]. On the other hand, emergency support is an important basis for the successful implementation of emergency response and disposal [29]. Meanwhile, it is also the core allocation mode of emergency resources in the process of emergency response [30]. As an emergency organization fulfilling the responsibility of emergency support, the government support institution mainly undertakes the functions of emergency resource invocation, transportation, distribution, on-site protection, blockade, and restriction. The government support institution also serves as the information channel and communication platform connecting the government rescue team to the social emergency organization. It can be concluded that the government support institution plays a key role as a bridge and medium in the process of emergency rescues.

It can be seen from the above discussion that emergency rescue cooperation inevitably involves coordination and cooperation between emergency rescue subjects. Meanwhile, the strategy selection of emergency rescue subjects will be influenced by other subjects [31,32]. At present, evolutionary game theory has also been applied and developed in the study of emergency management cooperation. Scholars describe and depict the cooperation and game relationship between emergency management organizations based on the strategy types and evolution process in emergency management [33]. The vertical emergency game between the upper and lower governments [34]; the horizontal emergency game in cross-regional government [35]; the game relationship between government, enterprises, and the public [36]; and the emergency cooperation game between the government and non-profit organizations [37] are important directions of the emergency cooperation game at the present stage. In addition, evolutionary game theory has made some progress in public health events [38], safety accidents [39], natural disasters [40], and other emergencies.

In terms of the study of the relationship between emergency subjects, the academic community is trying to explain the internal and external factors that affect the relationship interaction and strategy selection between emergency response organizations from the perspectives of reciprocity, reward, punishment, and third parties [41,42,43]. However, most existing studies regard relevant government departments with the same political intention and emergency target as a unified whole. The government departments are often proposed as the same subject of the game system. The above research does not discuss the strategic evolution among government organizations with different functions. To some extent, the decision-making characteristics and differences between various functional organizations in emergency management are ignored. Therefore, this study will introduce two directions of game selection between relevant government departments and social emergency organizations, emergency rescue organizations, and emergency support organizations.

The key point of this article is to study the dynamic process of the impact of the key parameters in emergency rescue cooperation, such as emergency capital stock, the implementation efficiency of emergency rescues, the effect coefficient of emergency cooperation, and the intensity coefficient of emergency support during emergency rescue cooperation by using the evolutionary game model so as to provide a theoretical reference for the mechanism design in the formulation of guidelines and emergency plans. The strategy selection modes for various organizations in the emergency rescue process will then be explored. The research results and conclusions have both theoretical and practical value for improving emergency rescue mechanisms and the effectiveness of emergency rescues.

## 3. Problem Description, Assumptions, and Game Model

### 3.1. Problem Description

At the present stage, the emergency rescue organizational model is gradually shifting from less interaction to full interaction between the government rescue team and the social emergency organization, with the government support institution providing all-round support in China. Various exchanges, interactions, and communication behaviors promote the diversification of cooperation modes, the expansion of the cooperation scale, and the further consolidation of the cooperation level among various emergency rescue subjects. As the main subject of emergency rescue responsibility, the government provides the main rescue force and coordinates the needs and resources of emergency responses. Further, the government will also promote the coordination and cooperation of various rescue organizations and the effective docking of emergency resources.

In the emergency rescue operations, there is not only a directive relationship between the vertical leadership of the state department to the local government but also a horizontal coordination interaction between the government rescue team, the government support institution, and the social emergency organization [44]. Among them, government rescue teams undertake emergency dispatching, rescue commands, search-and-rescue personnel, on-site isolation, and other rescue functions, which are the main command subjects and backbone of emergency rescues. Government support institutions undertake the support functions of logistics material support, power support, equipment support, communication support, oil support, order maintenance, and other support functions on the scene of emergency rescues, which are the resource support forces of emergency rescues. Social emergency organizations is the general term used for volunteer rescue teams, public welfare associations, charitable organizations, and other relevant organizations that participate in emergency rescues. They are mainly responsible for assisting government rescue teams in emergency rescues and cooperating with the government support institution to implement logistics support and other tasks. Social emergency organizations are important supplementary and auxiliary forces for emergency rescue work.

Overall, the cooperation strategies of emergency rescue subjects are influenced by both horizontal organizational cooperation and vertical administrative constraints [45]. This study focuses on the horizontal cooperation between the government rescue team, the government support institution, and the social emergency organization, and the administrative constraints of the vertical directive relationship are incorporated into the parametric hypothesis. This study will attempt to clarify and illustrate the evolution path of cooperative strategies of the government rescue team, the government support institution, and the social emergency organization in emergency rescues.

### 3.2. Assumptions and Parameters of the Game Model

**Figure 1:**
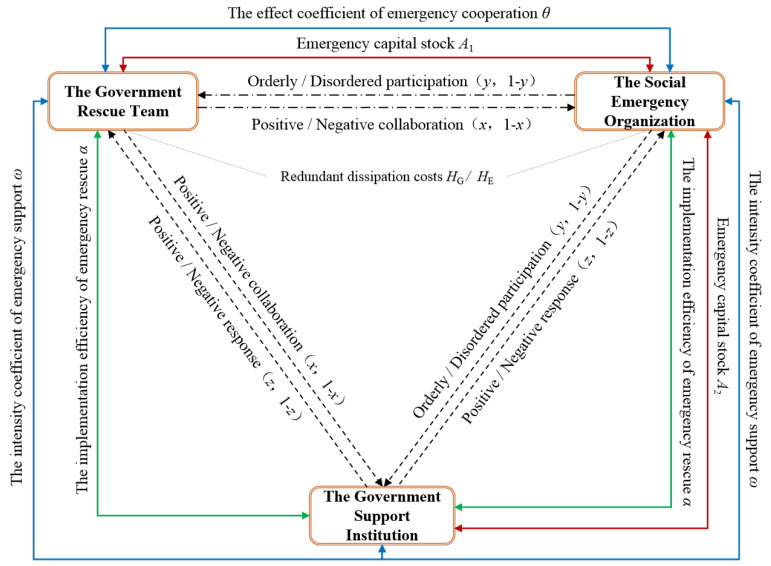
The game relationship between the government rescue team, the social emergency organization, and the government support institution.

Based on the game choice of government departments and social organizations, emergency rescue organizations, and emergency support organizations, this study constructed a tripartite evolutionary game model of the government rescue team, the social emergency organization, and the government support institution, as shown in Figure 1. In order to clarify the dynamic selection mechanism of the government rescue team in coordinating emergency rescue operations, the social emergency organization in participating in emergency rescue implementation, and the government support institution in implementing support measures, the following assumptions were made. Table 1 displays the definitions of the relevant parameters in the model.

**Assumption** **1.** *As the main subject of responsibility for emergency rescues, the government performs the function of unified commands, comprehensive coordination, and guiding the coordination and orderly participation of rescue organizations. On the one hand, under the functional division norms and administrative constraints of superior departments, the government rescue team will choose a positive collaboration strategy to improve rescue efficiency, reduce social losses caused by emergencies, and avoid secondary accident losses. At the same time, if the government rescue team does not have a clear understanding of the organizational scale, contact information, personnel quality, and equipment allocation of the social emergency organization, they will choose negative collaboration strategies in order to avoid unclear rescue commands and poor information transmission and to reduce the cost of emergency coordination. The probabilities of positive and negative collaboration strategies chosen by the government rescue team are x and 1 − x (0 ≤ x ≤ 1), respectively.*

**Assumption** **2.** *As an important supplement to the government emergency response system, the social emergency organization is a key force in assisting the government emergency system in managing emergencies and carrying out rescue operations. For better cooperation and coordination with the relevant government departments, the social emergency organization, mainly in the form of volunteer rescue teams, public welfare associations, and charitable organizations, will choose orderly participation strategies. At the same time, due to the limited sources of funds, organization patterns, and other issues, the social emergency organization will participate in the enthusiasm of emergency rescue operations. In addition, if good communication channels are not established with government departments, the orderly participation strategy in emergency rescue operations will cause the social emergency organization to pay more management costs. Therefore, the social emergency organization may choose an emergency rescue strategy of disordered participation. The probabilities of orderly and disordered participation strategies chosen by the social emergency organization are y and 1 − y (0 ≤ y ≤ 1), respectively.*

**Assumption** **3.** *In an emergency rescue, the government support institution will choose a positive response strategy under a certain vertical administrative constraint mechanism and political function norms. The government support institution is aware of all kinds of losses caused by emergencies and have a full understanding that emergency support can effectively improve the efficiency of emergency rescues. At the same time, due to the high cost of resources, information, and organization for emergency support, the government support institution may reduce the level of support to a certain extent and choose a negative response strategy. The probabilities of positive and negative response strategies chosen by the government support institution are z and 1 − z (0 ≤ z ≤ 1), respectively.*

**Assumption** **4.** *The government rescue team, the social emergency organization, and the government support institution are all bounded rationality in the emergency rescue cooperation evolutionary game model. Since the emergency rescue operations are implemented under the leadership of the emergency headquarters, there is obvious information asymmetry between government forces and social forces or between rescue forces and support forces. At the same time, the game strategies of the government rescue team, the social emergency organization, and the government support institution are randomly matched and repeated gaming.*

**Assumption** **5.** *Compared with the government alone managing emergencies, the participation of the social emergency organization in emergency rescue and disposal can give full play to the advantages of flexible organization and diversified services to improve emergency efficiency and reduce disaster losses. To some extent, the government’s emergency rescue costs will be reduced, but the government rescue team and the government support institution will also increase the coordination cost of the rescue because of the overall planning, guidance, and regulation of the social emergency organization to participate in emergency rescue operations. On the one hand, based on social capital theory [46,47], the government rescue team and the social emergency organization can form emergency capital stock based on trust relationships and institutional norms through joint plan preparation, daily emergency preparedness, emergency rescue drills, rescue cooperation in past accidents, and other modes [48]. The emergency capital stock based on information matching will also be formed between the government support institution and the social emergency organization through organizational information registration, information platform construction, resource type docking, and other methods. The emergency capital stock reflects the relationship between different emergency organizations and directly affects the cooperation and interaction of organizations in emergency rescues. A high emergency capital stock means that the corresponding rescue organizations could accrue lower costs in emergency rescue operations. On the other hand, when the social emergency organization participates in the emergency rescue operations, there will be problems, such as poor information communication and blocked docking channels, between the social emergency organization and the government departments due to the lack of an effective coordination mechanism [49]. It may evolve into the phenomenon of disordered “convergence” of the social emergency organization, forming the cooperation redundancy among emergency rescue organizations [50]. The government rescue team and the social emergency organization then need to pay the additional costs of rescue cooperation and coordination.*

**Assumption** **6.** *When the social emergency organization participates in emergency rescue operations, relevant government departments will give certain subsidies and compensation for the loss of the materials and equipment of social emergency rescue forces and for the cost of the life insurance of rescue workers. Meanwhile, relevant government departments will give appropriate rewards to the social emergency organization that participates in emergency rescue operations in an orderly manner. In addition, when summarizing emergency rescue operations and publicizing public welfare, relevant government departments will publicize the typical deeds of the social emergency organization with outstanding performance and active participation in emergency rescues. The positive public opinion orientation and good social atmosphere created will make the social emergency organization gain a certain reputation and benefits.*

**Assumption** **7.** *The effect coefficient of emergency cooperation θ is a variable used to measure the cooperation scale and tacit cooperation degree between the government rescue team and the social emergency organization. Under the positive coordination strategy of the government rescue team and the orderly participation strategy of the social emergency organization, a high-level θ means that there is a higher level of information transmission, resource sharing, and equipment sharing between the government rescue team and the social emergency organization. Meanwhile, when the effect coefficient of emergency cooperation is higher, the emergency organizations can effectively reduce the social development losses caused by emergencies, and the additional management costs paid by the government rescue team and the social emergency organization to achieve an orderly rescue.*

**Assumption** **8.** *The implementation efficiency of emergency rescue α is a variable used to evaluate the implementation effect of the overall emergency rescue operations under the limited input of emergency resources. Under the positive response strategy of the government support institution, if there is a high degree of matching and adaptability between emergency organizations in support categories and modes, the implementation efficiency of the emergency rescue will have a greater value. Meanwhile, a higher α indicates that the efficiency of emergency organizations in terms of search-and-rescue operations, medical treatment, evacuations, and the disposal of hazard sources is better. The government departments’ perception of the loss caused by emergencies and the additional management costs of dissipating cooperative redundancy will also be reduced.*

**Assumption** **9.** *The intensity coefficient of emergency support ω is a variable that reflects the completeness and investment intensity of financial support, power support, communication support, equipment support, logistics material support, order maintenance, oil support, and other support measures implemented by the government support institution. A high-level ω means that the government support institution has a large investment in fulfilling various support functions and providing financial support. At this time, the support costs of the government support institution and the compensation given by the governments to the social emergency organization will also increase accordingly. When the government support institution chooses a positive response strategy, the intensity coefficient of emergency support ω is set to 1.*

### 3.3. Payoff Matrix and Replication Dynamic Equations

Based on the game scenario of the government rescue team, the social emergency organization, and the government support institution under the cooperation mechanism of emergency rescues given by the research, the payoff matrix under different cooperation strategy combinations of the game subjects is summarized, as shown in Table 2 and Table 3.

#### 3.3.1. The Government Rescue Team

The expected benefit of the positive collaboration strategy selected by the government rescue team *E*_11_ is as follows:

(1) $$\begin{array}{l} E_{11}=yz\left[I-\left(C_{G}-A_{1}\right)-\left(1-\alpha \right)\left(1-\theta \right)L_{1}-\left(1-\alpha \right)\left(1-\theta \right)H_{G}\right]+y\left(1-z\right)\left[I-\left(C_{G}-A_{1}\right)-\left(1-\theta \right)L_{1}-\left(1-\theta \right)H_{G}\right]\\ +z\left(1-y\right)\left[I-C_{G}-\left(1-\alpha \right)L_{1}-\left(1-\alpha \right)H_{G}\right]+\left(1-y\right)\left(1-z\right)\left[I-C_{G}-L_{1}-H_{G}\right] \end{array}$$

The expected benefit of the negative collaboration strategy selected by the government rescue team *E*_12_ is as follows:

(2) $$E_{12}=yz\left(I-C_{G}-L_{1}-P_{G}\right)+y\left(1-z\right)\left(I-C_{G}-L_{1}-P_{G}\right)+z\left(1-y\right)\left(I-C_{G}-L_{1}-P_{G}\right)+\left(1-y\right)\left(1-z\right)\left(I-C_{G}-L_{1}-P_{G}\right)$$

The average expected benefit of the government rescue team’s collaboration strategy is:

(3) $$\bar{E_{1}}=xE_{11}+\left(1-x\right)E_{12}$$

The replication dynamic equation of the government rescue team’s collaboration strategy is:

(4) $$\begin{array}{l} F\left(x\right)=\frac{dx}{dt}=x\left(E_{11}-\bar{E_{1}}\right)=x\left(1-x\right)\left(E_{11}-E_{12}\right)=x\left(1-x\right)\\ \left[yA_{1}+P_{G}+\left(y\theta +z\alpha -yz\theta \alpha \right)L_{1}-\left(1-z\alpha \right)\left(1-y\theta \right)H_{G}\right] \end{array}$$

The derivation of *F*(*x*) is then obtained:

(5) $$F^{\prime }\left(x\right)=\left(1-2x\right)\left[yA_{1}+P_{G}+\left(y\theta +z\alpha -yz\theta \alpha \right)L_{1}-\left(1-z\alpha \right)\left(1-y\theta \right)H_{G}\right]$$

#### 3.3.2. The Social Emergency Organization

The expected benefit of the orderly participation strategy selected by the social emergency organization *E*_21_ is as follows:

(6) $$\begin{array}{l} E_{21}=xz\left[G+T+R-\left(C_{E}-A_{1}-A_{2}\right)-\left(1-\alpha \right)\left(1-\theta \right)H_{E}\right]+x\left(1-z\right)\left[\varpi \left(G+T\right)+R-\left(C_{E}-A_{1}\right)-\left(1-\theta \right)H_{E}\right]\\ +z\left(1-x\right)\left[G+T+R-\left(C_{E}-A_{2}\right)-\left(1-\alpha \right)H_{E}\right]+\left(1-x\right)\left(1-z\right)\left[\varpi \left(G+T\right)+R-C_{E}-H_{E}\right] \end{array}$$

The expected benefit of the disordered participation strategy selected by the social emergency organization *E*_22_ is as follows:

(7) $$E_{22}=xz\left(T-C_{E}\right)+x\left(1-z\right)\left(\varpi T-C_{E}\right)+z\left(1-x\right)\left(T-C_{E}\right)+\left(1-x\right)\left(1-z\right)\left(\varpi T-C_{E}\right)$$

The average expected benefit of the social emergency organization’s participation strategy is:

(8) $$\bar{E_{2}}=yE_{21}+\left(1-y\right)E_{22}$$

The replication dynamic equation of the social emergency organization’s participation strategy is:

(9) $$\begin{array}{l} G\left(y\right)=\frac{dy}{dt}=y\left(E_{21}-\bar{E_{2}}\right)=y\left(1-y\right)\left(E_{21}-E_{22}\right)=y\left(1-y\right)\\ \left[R+xA_{1}+zA_{2}+\left(z+\varpi -z\varpi \right)G-\left(1-x\theta \right)\left(1-z\alpha \right)H_{E}\right] \end{array}$$

The derivation of *G*(*y*) is then obtained:

(10) $$G^{\prime }\left(y\right)=\left(1-2y\right)\left[R+xA_{1}+zA_{2}+\left(z+\varpi -z\varpi \right)G-\left(1-x\theta \right)\left(1-z\alpha \right)H_{E}\right]$$

#### 3.3.3. The Government Support Institution

The expected benefit of the positive response strategy selected by the government support institution *E*_31_ is as follows:

(11) $$\begin{array}{l} E_{31}=xy\left[M_{S}-\left(C_{S}-A_{2}\right)-\left(1-\alpha \right)L_{2}\right]+x\left(1-y\right)\left[M_{S}-C_{S}-\left(1-\alpha \right)L_{2}-E\right]\\ +y\left(1-x\right)\left[M_{S}-\left(C_{S}-A_{2}\right)-L_{2}-E\right]+\left(1-x\right)\left(1-y\right)\left[M_{S}-C_{S}-L_{2}-E\right] \end{array}$$

The expected benefit of the negative response strategy selected by the government support institution *E*_32_ is as follows:

(12) $$E_{32}=xy\left(-\varpi C_{S}-L_{2}-P_{S}\right)+x\left(1-y\right)\left(-\varpi C_{S}-L_{2}-P_{S}\right)+y\left(1-x\right)\left(-\varpi C_{S}-L_{2}-P_{S}\right)+\left(1-x\right)\left(1-y\right)\left(-\varpi C_{S}-L_{2}-P_{S}\right)$$

The average expected benefit of the government support institution’s response strategy is:

(13) $$\bar{E_{3}}=zE_{31}+\left(1-z\right)E_{32}$$

The replication dynamic equation of the government support institution’s response strategy is:

(14) $$H\left(z\right)=\frac{dz}{dt}=z\left(E_{31}-\bar{E_{3}}\right)=z\left(1-z\right)\left(E_{31}-E_{32}\right)=z\left(1-z\right)\left[M_{S}+yA_{2}+P_{S}+x\alpha L_{2}-\left(1-\varpi \right)C_{S}-\left(1-xy\right)E\right]$$

The derivation of *H*(*z*) is then obtained:

(15) $$H^{\prime }\left(z\right)=\left(1-2z\right)\left[M_{S}+yA_{2}+P_{S}+x\alpha L_{2}-\left(1-\varpi \right)C_{S}-\left(1-xy\right)E\right]$$

Therefore, the replicated dynamic system of emergency rescue cooperation game can be obtained as follows:

(16) $$\left\{ \begin{array}{l} F\left(x\right)=x\left(1-x\right)\left[yA_{1}+P_{G}+\left(y\theta +z\alpha -yz\theta \alpha \right)L_{1}-\left(1-z\alpha \right)\left(1-y\theta \right)H_{G}\right]\\ G\left(y\right)=y\left(1-y\right)\left[R+xA_{1}+zA_{2}+\left(z+\varpi -z\varpi \right)G-\left(1-x\theta \right)\left(1-z\alpha \right)H_{E}\right]\\ H\left(z\right)=z\left(1-z\right)\left[M_{S}+yA_{2}+P_{S}+x\alpha L_{2}-\left(1-\varpi \right)C_{S}-\left(1-xy\right)E\right] \end{array}\right.$$

## 4. Strategic Stability Analysis of an Emergency Rescue Cooperation Evolutionary Game Model

### 4.1. Strategic Stability Analysis of the Government Rescue Team

When F(x)=0 and $F^{\prime }\left(x\right)<0$, the government rescue team has a stable probability of collaboration strategy selection. Further, when $z=z_{0}=-\frac{yA_{1}\;+\;P_{G}\;+\;y\theta L_{1}\;-\left(1\;-\;y\theta \right)H_{G}}{\alpha \left(1\;-\;y\theta \right)L_{1}\;+\;\alpha \left(1\;-\;y\theta \right)H_{G}}$ or $y=y_{0}=-\frac{P_{G}\;+\;z\alpha L_{1}\;-\left(1\;-\;z\alpha \right)H_{G}}{A_{1}\;+\;\theta \left(1\;-\;z\alpha \right)L_{1}\;+\;\theta \left(1\;-\;z\alpha \right)H_{G}}$, F(x) is equal to 0. The collaboration strategy adopted by the government rescue team in any mode is stable, and the strategy selection will not change with the evolution of the system.

When *z* > *z*_0_ or *y* > *y*_0_, $F^{\prime }\left(x\right)|{}_{x=0}>0$ and $F^{\prime }\left(x\right)|{}_{x=1}<0$ can be obtained. The government rescue team and the government support institution have the same emergency target, and the government rescue team is worried about the penalty loss, so there will not be “free-riding” behavior. The government rescue team tends to choose a positive collaboration strategy to strengthen the alliance and minimize the damage caused by emergencies. At the same time, additional emergency management costs paid by the government rescue team will be reduced, which will be more conducive to rescue target achievement. A positive collaboration is the stable strategy of the government rescue team. On the contrary, when *z* < *z*_0_ or *y* < *y*_0_, $F^{\prime }\left(x\right)|{}_{x=0}<0$ and $F^{\prime }\left(x\right)|{}_{x=1}>0$ can be obtained. The government rescue team realizes that the increasing investment and collaborative efforts will not effectively reduce accident loss or obtain more rescue benefits. Meanwhile, the government rescue team should pay more costs regarding coordination, organization, and commands in order to realize the orderly cooperation with the social emergency organization. The rescue cost of diversification and the rescue intention of blocks will bring greater losses. As a result, the government rescue team is more likely to choose a negative collaboration strategy.

**Figure 2:**
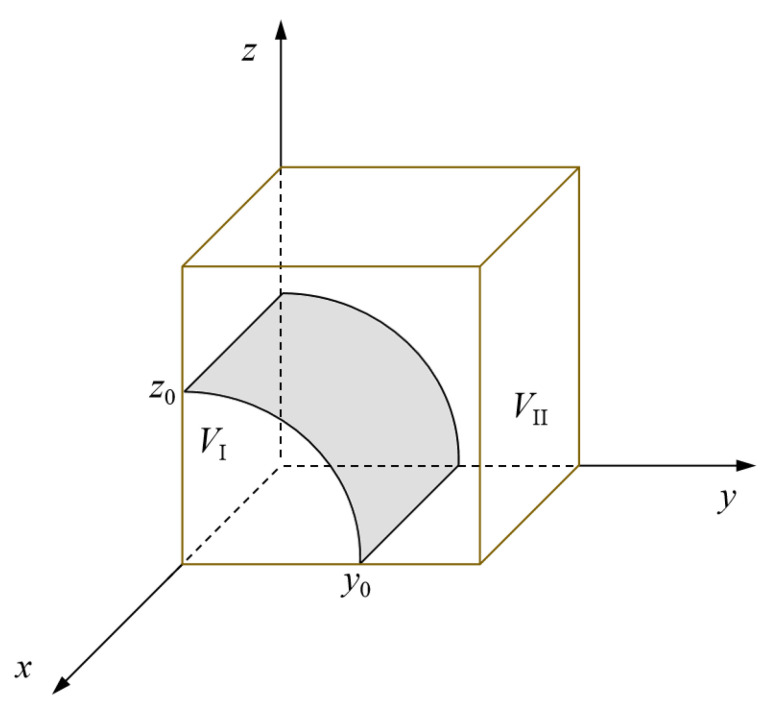
Evolutionary phase diagram of the government rescue team’s strategy.

To sum up, the probability that the government rescue team chooses a positive collaboration strategy is directly proportional to the probability that the social emergency organization chooses an orderly participation and the probability that the government support institution chooses a positive response. Further, the evolutionary phase diagram of the government rescue team’s strategy is drawn, as shown in Figure 2. The probability that the government rescue team chooses a negative collaboration strategy is represented by the volume *V*_I_ of region I. Likewise, the probability that the government rescue team chooses a positive collaboration strategy is represented by the volume *V*_II_ of region II.

According to the phase diagram, the value of *z*_0_ is inversely proportional to the probability that the government rescue team chooses a positive collaboration strategy. A greater *z*_0_ indicates that the government rescue team is less likely to choose a positive collaboration strategy. Taking partial derivatives of *z*_0_ with respect to *A*_1_, *P_G_*, *L*, *θ*, *α*, and *H_G_*, it can be known that *z*_0_ is a decreasing function with respect to *A*_1_, *P_G_*, *L*, *θ*, and *α* and an increasing function with respect to *H_G_*. With the increase of the perception of the loss caused by emergencies and the perception of the benefits to be gained, the government rescue team will choose a positive collaboration strategy. The probability *V*_II_ that the government rescue team chooses a positive strategy will increase by improving *A*_1_, *P_G_*, *L*, *θ*, and *α* or decreasing *H_G_*. This suggests that emergency trust capital and emergency regulation capital, carrying out supervision and inspection, enhancing tacit cooperation, and strengthening support for rescue organizations are all important measures to encourage the government rescue team to choose a positive collaboration strategy.

### 4.2. Strategic Stability Analysis of the Social Emergency Organization

When G(y)=0 and $G^{\prime }\left(y\right)<0$, the social emergency organization has a stable probability of participation strategy selection. Further, when $z=z_{0}=-\frac{R\;+\;xA_{1}\;+\;\varpi G\;-\left(1\;-\;x\theta \right)H_{E}}{A_{2}\;+\left(1\;-\;\varpi \right)G\;+\;\alpha \left(1\;-\;x\theta \right)H_{E}}$ or $x=x_{0}=-\frac{R\;+\;zA_{2}\;+\left(z\;+\;\varpi \;-\;z\varpi \right)G\;-\left(1\;-\;z\alpha \right)H_{E}}{A_{1}\;+\;\theta \left(1\;-\;z\alpha \right)H_{E}}$, G(y) is equal to 0. The participation strategy adopted by the social emergency organization in any mode is stable, and the strategy selection will not change with the evolution of the system.

When *z* > *z*_0_ or *x* > *x*_0_, $G^{\prime }\left(y\right)|{}_{y=0}>0$ and $G^{\prime }\left(y\right)|{}_{y=1}<0$ can be obtained. The social emergency organization participating in the emergency rescue will receive certain a loss compensation from the government and additional reward income. With the support of the government rescue team and the government support institution, the social emergency organization can save some rescue costs through the emergency capital stock. Therefore, orderly participation is a stable strategy for social emergency organization. On the contrary, when *z* < *z*_0_ or *x* < *x*_0_, $G^{\prime }\left(y\right)|{}_{y=0}<0$ and $G^{\prime }\left(y\right)|{}_{y=1}>0$ can be obtained. The social emergency organization will receive less compensation and incentives and pay more administrative costs to offset the redundancy of emergency rescue cooperation. In other words, the social emergency organization has a low probability of benefiting from rescue cooperation. At this point, the social emergency organization will be more inclined to choose a disordered participation strategy.

**Figure 3:**
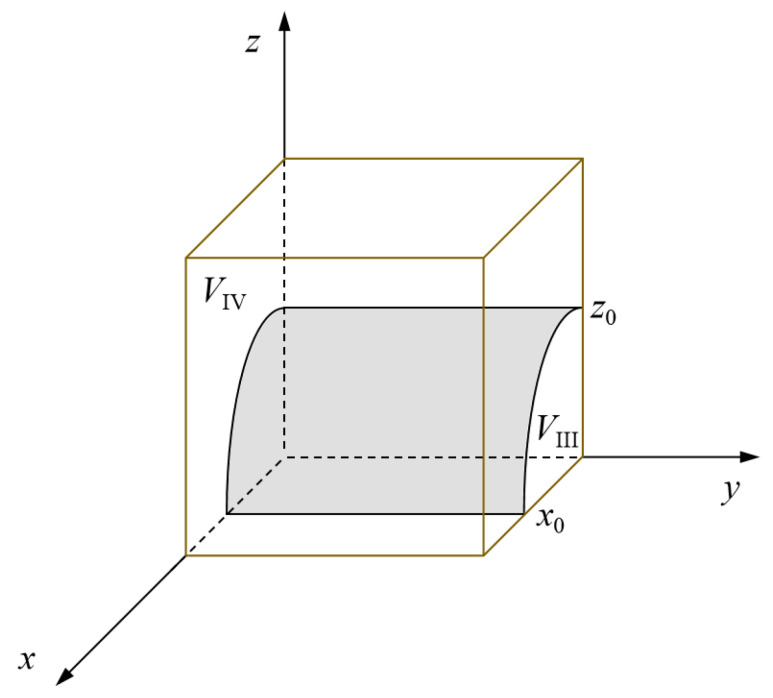
Evolutionary phase diagram of the social emergency organization’s strategy.

In conclusion, with an increasing probability that a government rescue team chooses a positive collaboration strategy and that the government support institution chooses a positive response strategy, the probability that the social emergency organization chooses an orderly participation strategy will gradually increase. Accordingly, the evolutionary phase diagram of the social emergency organization’s strategy can be obtained (see Figure 3). The volume *V*_III_ of region III represents the probability that the social emergency organization selects a disordered participation strategy. Likewise, the volume *V*_IV_ of region IV represents the probability that the social emergency organization selects an orderly participation strategy.

As shown in Figure 3, the smaller *z*_0_ is, the greater the probability that the social emergency organization chooses an orderly participation strategy. On the contrary, the larger *z*_0_ is, the smaller the probability that the social emergency organization chooses an orderly participation strategy. Taking the partial derivatives of *z*_0_ with respect to *R*, *A*_1_, *A*_2_, *G*, *θ*, *α*, *ω*, and *H_E_*, it can be known that the value of *z*_0_ is a decreasing function with respect to *R*, *A*_1_, *A*_2_, *G*, *θ*, *α*, and *ω* and an increasing function with respect to *H_E_*. For the social emergency organization, *z*_0_ will decrease with the increase of various benefits (social reputation benefits and government reward benefits) and additional costs paid by disorderly participation. The social emergency organization will be more inclined to choose an orderly participation strategy. When *R*, *A*_1_, *A*_2_, *G*, *θ*, *α*, and *ω* are strengthened or *H_E_* is decreased, the probability that the social emergency organization chooses an orderly participation strategy *V*_IV_ will increase accordingly. Therefore, strengthening inter-organizational contact, establishing coordination and service platforms, improving the level of cooperation in the emergency preparedness stage, strengthening guidance and guarantee, and increasing the recognition, publicity, and reward support for the social emergency organization will all effectively promote the orderly participation of the social emergency organization in emergency rescue operations.

### 4.3. Strategic Stability Analysis of the Government Support Institution

When H(z)=0 and $H^{\prime }\left(z\right)<0$, the government support institution has a stable probability of response strategy selection. Further, when $x=x_{0}=-\frac{M_{S}\;+\;yA_{2}\;+\;P_{S}\;-\left(1\;-\;\varpi \right)C_{S}\;-\;E}{\alpha L_{2}\;+\;yE}$ or $y=y_{0}=-\frac{M_{S}\;+\;P_{S}\;+\;x\alpha L_{2}\;-\left(1\;-\;\varpi \right)C_{S}\;-\;E}{A_{2}\;+\;xE}$, H(z) is equal to 0. The response strategy adopted by the government support institution in any mode is stable, and the strategy selection will not change with the evolution of the system.

When *x* > *x*_0_ or *y* > *y*_0_, $H^{\prime }\left(z\right)|{}_{z=0}>0$ and $H^{\prime }\left(z\right)|{}_{z=1}<0$ can be obtained. The government support institution should avoid the punishment and accountability loss of superior departments caused by the failure to fulfill their duties. At the same time, in order to achieve the rescue target of reducing the loss caused by emergencies and secondary accidents, the probability that the government support institution chooses a positive response strategy will gradually converge to 1. On the contrary, when *x* < *x*_0_ or *y* < *y*_0_, $H^{\prime }\left(z\right)|{}_{z=0}<0$ and $H^{\prime }\left(z\right)|{}_{z=1}>0$ can be obtained. At this time, the government support institution needs to pay additional costs to maintain a certain efficiency of emergency rescue implementation. Meanwhile, the emergency capital stock fails to form beneficial resource supplements for the government support institution, and the support given to the social emergency organization will decrease. Therefore, the government support institution will form a stable strategy of a negative response.

**Figure 4:**
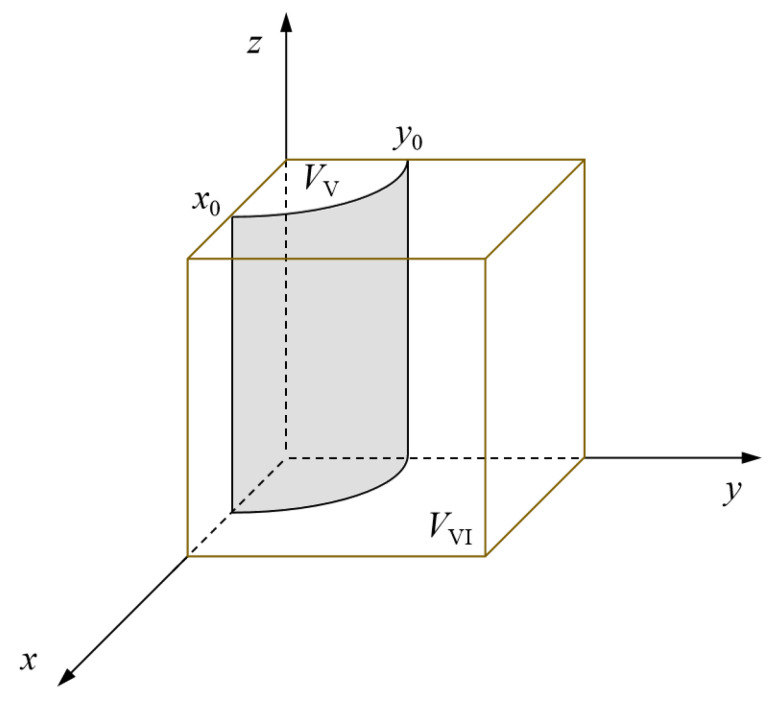
Evolutionary phase diagram of the government support institution’s strategy.

In conclusion, the probability that the government support institution chooses a positive response strategy is directly proportional to the probability that the social emergency organization chooses orderly participation and the probability that the government rescue team chooses a positive collaboration. Figure 4 shows the evolutionary phase diagram of the government support institution’s strategy. Among them, the probability that the government support institution chooses a negative response strategy is represented by the volume *V*_V_ of region V. Likewise, the probability that the government support institution chooses a positive response strategy is determined by the volume *V*_VI_ of region VI.

As shown in Figure 4, the value of *x*_0_ is inversely proportional to the probability that the government support institution chooses a positive response strategy. Taking partial derivatives of *x*_0_ with respect to *M_S_*, *A*_2_, *P_S_*, *L*_2_, *α*, *ω*, *C_S_*, and *E*, it can be known that the value of *x*_0_ is a decreasing function with respect to *M_S_*, *A*_2_, *P_S_*, *L*_2_, *α*, and *ω* and an increasing function with respect to *C_S_* and *E*. For the government support institution, with the increase of reward and compensation benefits, and possible punishment loss and accountability loss by superior governments, the government support institution tends to choose a positive response strategy. Therefore, improving *M_S_*, *A*_2_, *P_S_*, *L*_2_, *α*, and *ω* or decreasing *C_S_* and *E* are the main means to increase the value of *V*_VI_. On the one hand, internal and external coordination should be strengthened to enhance the action willingness of the government support institution by improving guidance and supervision. On the other hand, we should establish an integrated emergency support mechanism and give full play to the important supporting role of emergency support for the implementation of emergency rescues.

## 5. Simulation Analysis of Emergency Rescue Cooperation Game System

The influence of different parameters and different initial strategies on the strategy selection of game subjects has been discussed and proved. Based on the results of strategic stability analysis of each game subject, the influence mechanism of *P_G_*, *R*, *G*, *H_G_*, *H_E_*, *L*_1_, *L*_2_, *C_S_*, *M_S_*, *P_S_*, and *E* on the strategy selection of the game subject is as follows. Firstly, *P_G_* and *L*_1_ have a positive impact on the government rescue team’s choice of a positive collaboration strategy, and *H_G_* has a negative impact. Secondly, *R* and *G* have a positive response to an orderly participation strategy of social emergency organization, and *H_E_* has a negative response. Thirdly, *M_S_*, *P_S_*, and *L*_2_ have a positive influence on the positive response strategy of the government support institution, while *C_S_* and *E* have a negative influence.

However, this article has not conducted in-depth analysis on the influence mechanism of different game hypothesis parameters on the evolution of the game system. Therefore, the dynamic evolution process of strategy selection among the government rescue team, the social emergency organization, and the government support institution will be simulated. The effects of emergency capital stocks *A*_1_ and *A*_2_, the effect coefficient of emergency cooperation *θ*, the implementation efficiency of emergency rescue *α*, and the intensity coefficient of emergency support *ω* on the evolution of the emergency rescue cooperation game system will be investigated. Combined with the data from the actual situation of emergency rescues in China, the initial values of each parameter in the model are given. Assume that *θ* = 0.3, *α* = 0.3, *ω* = 0.5, *H_G_* = 15, *L*_1_ = 15, *P_G_* = 8, *H_E_* = 15, *G* = 2, *R* = 2, *A*_1_ = 3, *A*_2_ = 3, *C_S_* = 15, *L*_2_ = 15, *P_S_* = 5, *M_S_* = 2, and *E* = 5. Meanwhile, the initial state of the emergency rescue cooperation game system is set as (0.5,0.5,0.5).

### 5.1. The Effect of A_1_ and A_2_ on the Evolution of the Emergency Rescue Cooperation Game System

**Figure 5:**
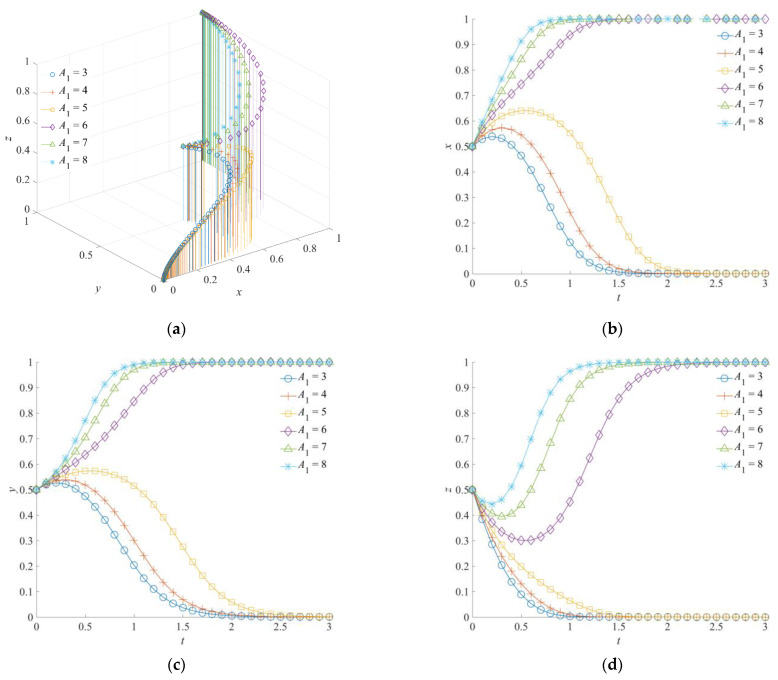
Evolution trajectory of the game system under different emergency capital stocks between the government rescue team and the social emergency organization. (**a**) The influence of *A*_1_ on the evolution of the game system. (**b**) The influence of *A*_1_ on the evolution of the government rescue team. (**c**) The influence of *A*_1_ on the evolution of the social emergency organization. (**d**) The influence of *A*_1_ on the evolution of the government support institution.

**Figure 6:**
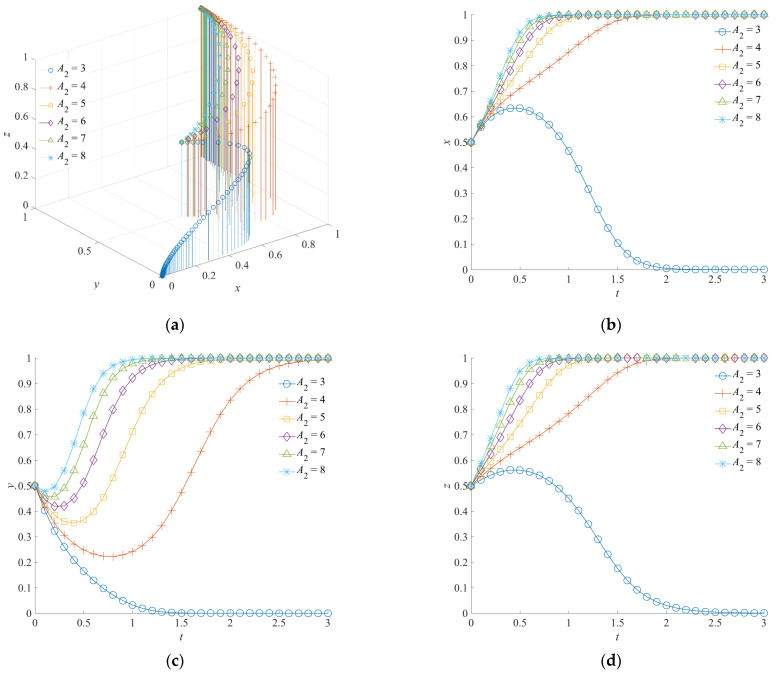
Evolution trajectory of the game system under different emergency capital stocks between the government support institution and the social emergency organization. (**a**) The influence of *A*_2_ on the evolution of the game system. (**b**) The influence of *A*_2_ on the evolution of the government rescue team. (**c**) The influence of *A*_2_ on the evolution of the social emergency organization. (**d**) The influence of *A*_2_ on the evolution of the government support institution.

The emergency capital stocks *A*_1_ and *A*_2_ are set as 3, 4, 5, 6, 7, and 8, respectively. The corresponding evolutionary track of the emergency rescue cooperation game system is shown in Figure 5 and Figure 6, respectively. When the emergency capital stock *A*_1_ between the government rescue team and the social emergency organization or the emergency capital stock *A*_2_ between the government support institution and the social emergency organization increases, the game system of the emergency rescue cooperation will eventually form the evolutionarily stability strategy (1,1,1).

To be specific, when the emergency capital stock *A*_1_ is high, the government rescue team, the social emergency organization, and the government support institution will evolve into a positive collaboration strategy, an orderly participation strategy, and a positive response strategy, respectively. A larger level of *A*_1_ means that the convergence rate of the evolution path will be faster. When *A*_1_ is low, although the government rescue team and the social emergency organization tend to choose the collaboration strategy and the orderly participation strategy, respectively, at the initial stage of evolution, the game system will eventually evolve toward (0,0,0). Furthermore, when the emergency capital stock *A*_2_ is high, positive collaboration, orderly participation, and a positive response are still the stable strategy combination of the system evolution. Similarly, a higher level of *A*_2_ will promote faster convergence of the system towards (1,1,1). At the same time, with the insufficient emergency capital stock *A*_2_, although the government rescue team and the government support institution tend to choose a positive collaboration strategy and a positive response strategy, respectively, due to the functional requirements and mission incentives, the game system will also evolve toward (0,0,0) at the final stage of evolution.

As can be seen from the above, the government rescue team and the government support institution are important driving forces for each other to choose a positive strategy. On the one hand, when the government rescue team or the government support institution chooses a positive strategy, it will be the self-disciplined choice of the other subject to perform the emergency rescue function according to the requirements of the superior department. On the other hand, when the emergency capital stock of the social emergency organization is insufficient, the government rescue team or the government support institution will choose a positive strategy in advance to support the effective implementation of the emergency rescue operations.

### 5.2. The Effect of θ on the Evolution of the Emergency Rescue Cooperation Game System

**Figure 7:**
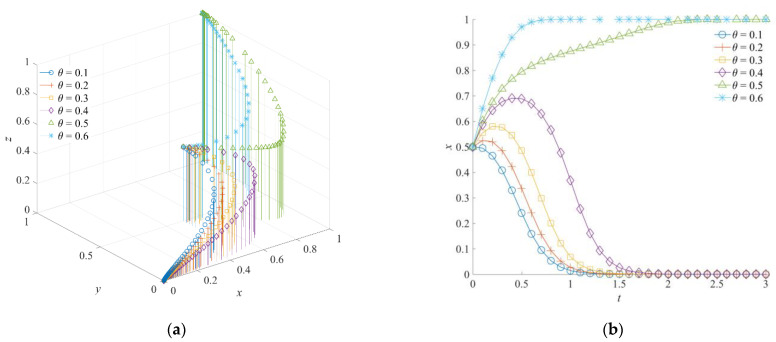

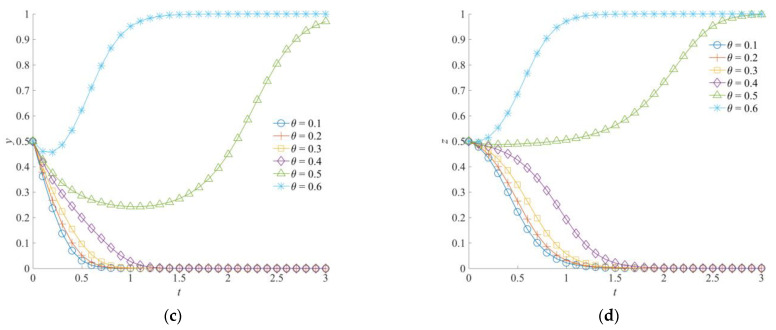
Evolution trajectory of the game system under different effect coefficients of emergency cooperation. (**a**) The influence of *θ* on the evolution of the game system. (**b**) The influence of *θ* on the evolution of the government rescue team. (**c**) The influence of *θ* on the evolution of the social emergency organization. (**d**) The influence of *θ* on the evolution of the government support institution.

The effect coefficient of emergency cooperation *θ* is set as 0.1, 0.2, 0.3, 0.4, 0.5, and 0.6, respectively. The value of *θ* corresponds to a 3-level intensity of low (0.1, 0.2), medium (0.3, 0.4), and high (0.5, 0.6). Under different effect coefficients of emergency cooperation, the obtained system evolution results are shown in Figure 7. Since *θ* is a variable describing the tacit cooperation between rescue forces, when the effect coefficient of emergency cooperation has a high strength, the strategy selection of each game subject will evolve towards positive collaboration, orderly participation, and a positive response. When the emergency for the cooperation effect coefficient has a high intensity, the tripartite game strategy evolves toward a (1,1,1) combination of convergence. Moreover, when the effect coefficient of emergency cooperation is at a medium and low intensity, the emergency rescue cooperation game system will converge to a (0,0,0) strategy combination.

When the emergency cooperation effect between the government rescue team and the social emergency organization is higher, the sharing level of information, resources, and equipment is better, which can effectively avoid problems, such as poor information communication and blocked docking channels. On the one hand, a high-level *θ* can reduce the uncertainty of cooperation between rescue forces in the process of emergency rescues and offset the cost of organization, management, and coordination. On the other hand, a high-level *θ* can improve the systematic and holistic level of emergency rescue implementation and reduce the social development losses caused by emergencies. Meanwhile, it can be seen in Figure 7 that the social emergency organization produces an indecisive choice of strategy. However, with the continuous evolution of the government rescue team choosing a positive collaboration strategy, the awareness of orderly participation of the social emergency organization is driven. The positive situation of emergency rescue cooperation is then finally formed. This indicates that the good cooperation between the government rescue team and the social emergency organization is the support and guarantee for improving the enthusiasm of the social emergency organization to participate in emergency rescues in an orderly manner. In addition, the government support institution will temporarily choose a negative response strategy due to their incomplete cognition of rescue practice. However, since the government rescue team drives the orderly rescue implementation of the social emergency organization, which increases the expected benefits of the government support institution for emergency rescues, the government support institution will choose the direction of a positive response strategy evolution.

### 5.3. The Effect of α on the Evolution of the Emergency Rescue Cooperation Game System

**Figure 8:**
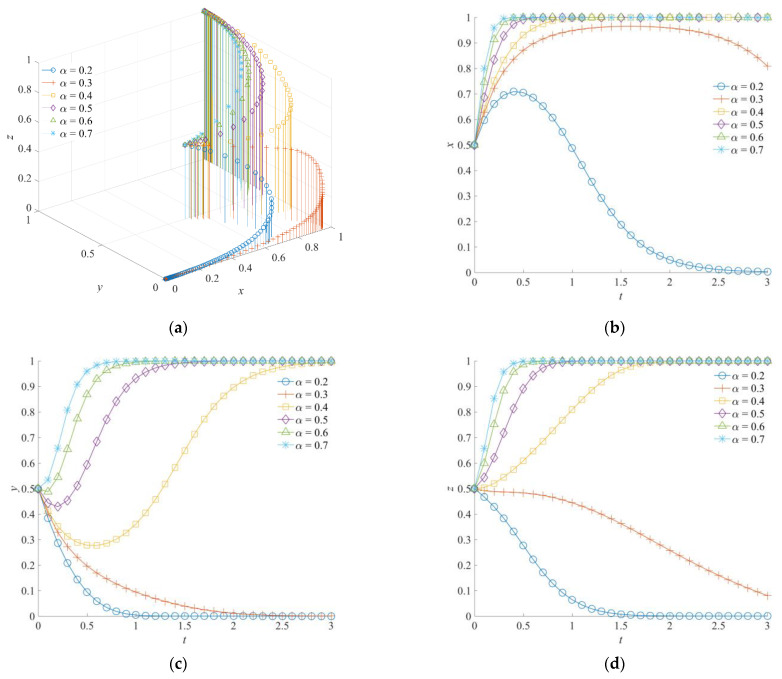
Evolution trajectory of the game system under different implementation efficiencies of emergency rescues. (**a**) The influence of *α* on the evolution of the game system. (**b**) The influence of *α* on the evolution of the government rescue team. (**c**) The influence of *α* on the evolution of the social emergency organization. (**d**) The influence of *α* on the evolution of the government support institution.

The implementation efficiency of emergency rescue *α* is set as low (0.2, 0.3), medium (0.4, 0.5), and high (0.6, 0.7). The evolution results of the corresponding emergency rescue cooperation game system analyzed by simulation are shown in Figure 8. The implementation efficiency of emergency rescues can represent the practice effect of emergency rescue operations. It is a parameter used to measure the support level of the government support institution for the emergency rescue force. When the implementation efficiency of an emergency rescue is low, the game subjects will evolve to a negative collaboration strategy, a disorderly participation strategy, and a negative response strategy. Increasing the implementation efficiency of emergency rescues will encourage the government rescue team to choose a positive collaboration strategy, the social emergency organization to choose an orderly participation strategy, and the government support institution to choose a positive response strategy, and the game system will finally form the strategy combination of (1,1,1). When the implementation efficiency of the emergency rescue is higher, the adaptability and matching degree between the government support institution and the emergency rescue forces in various measures of emergency support is better. A high level of *α* can improve the effectiveness of various rescue operations, such as search-and-rescue personnel, dangerous situation disposal, and medical treatment. In other words, the improvement of *α* will reduce the time required for emergency rescue and reduce the expenditure of various emergency rescue costs. Meanwhile, losses caused by secondary accidents and chain disasters can be avoided to a certain extent.

In addition, it should be noted that, in the process of improving the implementation efficiency of emergency rescues, although the government rescue team and the social emergency organization will eventually choose the strategy of positive collaboration and orderly participation, the evolution process is different. For the government rescue team, both it and the government support institution belong to the administrative system, which can form tacit cooperation more effectively and have consistent value orientation in the emergency rescue. Therefore, in the early stage of the system evolution, the government rescue team turns to a positive cooperation strategy at a faster speed, which promotes the convergence of the game system toward (1,1,1). For the social emergency organization, its strategy selection shows an evolutionary trend of disorder first and then order, which occurs with the continuous improvement of the probability that the government rescue teams choose a positive collaboration strategy. This indicates that the social emergency organization plays a follower role in the game system.

### 5.4. The Effect of ω on the Evolution of the Emergency Rescue Cooperation Game System

**Figure 9:**
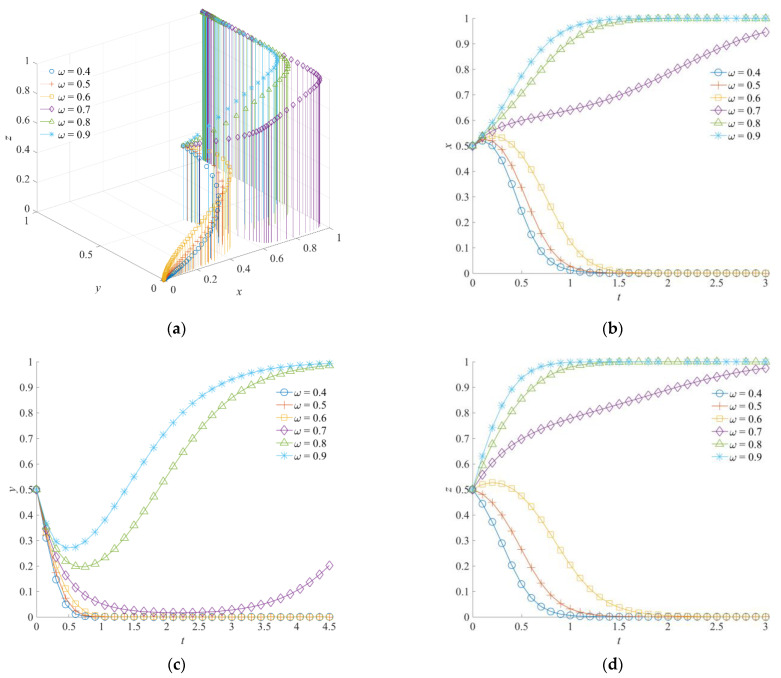
Evolution trajectory of the game system under different intensity coefficients of emergency support. (**a**) The influence of *ω* on the evolution of the game system. (**b**) The influence of *ω* on the evolution of the government rescue team. (**c**) The influence of *ω* on the evolution of the social emergency organization. (**d**) The influence of *ω* on the evolution of the government support institution.

Figure 9 shows the evolutionary trajectory of the emergency rescue cooperation game system under different intensity coefficients of emergency support *ω*. Among them, the *ω* is set as 0.4, 0.5, 0.6, 0.7, 0.8, and 0.9, respectively. By comparing the strategy simulation curves of each game subject, it can be seen that the intensity coefficient of emergency support not only affects the probability of the game subject forming a certain strategy, but also has an influence on the convergence speed of the game subject’s strategy selection.

Specifically, when the intensity coefficient of emergency support is low, the government rescue team realizes that the probability of the government rescue team’s strategy selection will gradually converge to 0 even if the government increases the input. It is not more effective to reduce emergency losses or gain more rescue benefits. With the improvement of ω, the initiative and enthusiasm of the government rescue team choosing a positive collaboration strategy are improved. The stable evolution strategy will experience a process from negative collaboration to positive collaboration. At the same time, the government rescue team is more decisive in the process of strategy evolution, which shows that the convergence speed to a stable strategy is faster than that of other game subjects. However, the convergence rate changes slightly with the further improvement of *ω*. When the intensity coefficient of emergency support *ω* is at a high level, the social emergency organization could obtain higher rewards and compensation from government departments. The social emergency organization tends to choose an orderly participation strategy. Instead, in the early stage of evolution, the social emergency organization forms a tendency to evolve toward a disordered participation strategy. With the increase of *ω*, the duration of its evolution to an orderly strategy is gradually shortened and the evolution speed of a stable strategy is gradually accelerated. The impact of *ω* on the strategy selection of the government support institution is more direct. When ω is high, although the government support institution tends to choose the negative strategy at the initial stage of evolution, they will turn to a positive response strategy driven by other game subjects. At the same time, the probability that the government support institution chooses a positive response strategy will increase as *ω* increases.

Meanwhile, it can be concluded that the improvement of the intensity coefficient of emergency support has a significantly higher impact on the strategic change of the government rescue team than that of the social emergency organization. This indicates that the cooperation between the government rescue team and the government support institution is more intimate. Although the strategy selection of the social emergency organization is stable under a high intensity coefficient of emergency support *ω*, the evolution process also experiences a fluctuation course from a disorderly participation strategy to an orderly participation strategy, which further verifies the conclusion obtained above.

## 6. Discussion

The effective implementation of emergency disposal and rescue needs coordination and cooperation among different subordinates and different levels of emergency organizations. The evolutionary game model of emergency rescue cooperation is constructed by summarizing the categories, characteristics, and behaviors of the participants in emergency rescues. This article introduces evolutionary game theory into emergency rescue cooperation, which is an attempt to describe the cooperative game relationship among the government rescue team, the social emergency organization, and the government support institution. The model sets up the emergency capital stock, the redundant dissipative cost, the implementation efficiency of emergency rescues, the effect coefficient of emergency cooperation, and the intensity coefficient of emergency support, which broadens the perspective and scope of describing the emergency rescue cooperation mode. The model assumptions and parameter settings in this article are derived from a consistent description of China’s emergency rescue practice, but the research results still have a certain practical reference value for governments around the world to improve the effectiveness of emergency rescue.

According to the simulation results of the emergency rescue cooperation game system, although the three subjects of the game—the government rescue team, the social emergency organization, and the government support institution—have an evolutionary trend to the opposite direction of the strategy in a certain period of time, they will eventually converge to (1,1,1) or (0,0,0). This is because each game subject has the same political demands and rescue targets in the emergency rescue cooperation, so each game subject has a similar motivation to choose a strategy. At the same time, the government rescue team as the leader of the rescue operations often has the fastest strategy evolution speed while the social emergency organization follows the strategy evolution path. The government rescue team and the government support institution bring more value to the social emergency organization through a positive strategy, which can promote the social emergency organization to choose orderly participation in emergency rescue operations. Therefore, on the whole, the evolution of the emergency rescue cooperation game model shows the characteristics of “mobilization-coordination” rather than “pressure-compulsion”.

The cost paid in emergency rescues is an important factor affecting the formation of an emergency rescue cooperation mechanism. Higher organization costs, human costs, and capital costs of each game subject in an emergency rescue are not conducive to the formation of a positive and orderly emergency rescue cooperation mode among the game subjects. At the same time, in the daily emergency preparedness, the emergency capital stock formed by trust relationships, information matching, and institutional norms among game subjects can effectively reduce the cost of emergency rescue operations. The evolutionarily stable strategy of the game system tends toward positive collaboration, orderly participation, and a positive response.

An emergency rescue response and the implementation of emergency rescue operations are important challenges to the situation of emergency resources owned by all game subjects. Strengthening the reduction and supplement of the consumption of emergency resources represents necessary support for the evolution of the emergency rescue cooperation game system toward a positive strategy. Specifically, under the vertical administrative constraints of the superior government, the rewards of a positive strategy and the punishment of a negative strategy will improve the probability of the government rescue team’s and the government support institution’s respective positive response strategies. The government’s compensation for loss, publicity, and commendation are effective measures to encourage the social emergency organization to choose an orderly participation strategy.

When changing the parameter values of the emergency capital stock, the implementation efficiency of emergency rescues, the effect coefficient of emergency cooperation, and the intensity coefficient of emergency support (although a lower parameter value) will encourage the government rescue team and the government support institution to evolve toward positive strategies, and the government organization will turn to a passive strategy due to a lack of investment. In other words, for government organizations with limited emergency resources, the emergency cost input scheme with an average distribution of all parameters is not the optimal solution to improve the efficiency of emergency rescues. In contrast, since an emergency rescue is a public welfare activity, it is easier to achieve a cooperation target when all subjects put limited emergency resources into a control plan that matches the key variables of the scene.

## 7. Suggestions and Implications

According to the above conclusions, this research puts forward countermeasures and suggestions to strengthen the mechanism construction of emergency rescue cooperation from six aspects with consideration of the related factors influencing emergency rescue cooperation.

(1) Strengthening the formulation of emergency plans and the implementation of emergency drills: Daily preparation for emergency rescues is an important means to enhance the emergency capital stock among emergency organizations, which will effectively reduce the cost of organization, coordination, and standardization. It can promote the close cooperation, rapid response, and orderly implementation of emergency rescue operations. On the one hand, the participation of the social emergency organization in emergency rescues should be taken as the key content of the emergency plan. At the same time, the coordination of emergency plans at all levels is needed to ensure a rapid response and the efficient linkage of emergency rescue operations. On the other hand, it is necessary to integrate emergency organizations and coordinate multiple forms of emergency preparedness. Through the sharing of training sites, joint emergency drills, rescue operations training, and other modes, emergency rescue preparedness and actual combat capabilities will be effectively improved. In addition, the emergency plan should be quickly improved and amended according to actual combat and drills to achieve a continuous updating of emergency preparedness.
(2) Establishing a comprehensive emergency information and service platform: The government should establish a comprehensive service platform for social emergency organizations to participate in emergency rescues to apply a comprehensive coordination function of emergency management. This platform could build a bridge of cooperation and communication between government functional departments and social emergency organizations. The efficiency of emergency rescues will then be improved. On the one hand, it is necessary to coordinate and guide social emergency organizations to quickly submit relevant information to the platform, to strengthen the government’s knowledge of the scale and situation of social emergency organizations. The government’s daily guidance and assignment will be carried out smoothly. On the other hand, it is necessary to expand the port of the emergency platform and unite all functional departments for access. It is also crucial to strengthen information disclosure and quickly push emergency information, such as disaster notification and rescue needs, in order to ensure unimpeded information and coordination among emergency organizations.
(3) Strengthening the overall deployment of emergency forces and the coordination of emergency commands: From the practice of emergency rescues, the scale of emergency forces and the emergency command system are important links to achieve emergency rescue targets. Promoting the coordinated deployment of various emergency forces and a complete emergency command structure will directly reduce the cost of emergency rescues and help improve the level of emergency rescue cooperation. On the one hand, it is necessary to promote the connection of different emergency forces and form a rescue system, with a government rescue team as the core and multiple emergency organizations as support. On the other hand, the flat construction of an emergency rescue command structure can lead to full coordination between on-site disposal and remote dispatching. Outstanding problems, such as unclear emergency rescue commands, poor command transmissions, and belated information feedback, will also be avoided.
(4) Promoting integrated implementation of emergency support and overall allocation of emergency resources: As the emergency resources are multifarious and diverse, the repeated allocation and disorderly supply of emergency resources occur easily. Improving the efficiency of emergency rescues and the intensity coefficient of emergency support will promote the evolution of the emergency rescue cooperation game system to a positive strategy combination. Therefore, it is necessary to strengthen the matching degree among government support institutions, the government rescue team, and the social emergency organization in the mode of emergency support, to achieve accurate and efficient emergency rescue operations. Firstly, the unified dispatch of emergency rescue equipment should be strengthened to realize the sharing of rescue equipment among emergency organizations. Secondly, emergency measures, such as on-site vigilance, road control, and evacuation, should be strengthened to achieve a smooth and orderly rescue scene. Thirdly, we should highlight the speed and accuracy of material support and establish a process mechanism of storage, transportation, and distribution. Fourthly, it is necessary to enhance the degree of localized expertise of emergency rescue organizations in first response and follow-up logistics management.
(5) Improving the policy system for social emergency organizations to participate in emergency rescues: A complete policy system will effectively reduce the emergency costs of social emergency organizations participating in emergency rescue operations. It will encourage and promote the emergency rescue cooperation game system to approach a positive strategy combination. Specifically, on the one hand, it is necessary to clarify the functional orientation of social emergency organizations and establish and improve the management methods and implementation procedures for social emergency organizations to participate in emergency rescues. On the other hand, incentive measures for social emergency organizations to participate in emergency rescues should be enriched. It is necessary to supplement the consumption and expenditure of social emergency organizations in rescue operations and establish an effective expropriation and compensation mechanism.
(6) Optimizing the supervision and reward mechanism: An effective reward and punishment mechanism is important to improve the performance of emergency rescues. On the one hand, superior government departments should strengthen the supervision and evaluation of emergency rescue operations and hold the relevant departments responsible if they fail to perform. Further, it will encourage the government rescue team to choose a positive cooperation strategy and government support institutions to choose a positive response strategy. On the other hand, the typical deeds of social emergency organizations should be widely publicized by means of emergency summaries, public welfare commendations, and emergency documentaries. Meanwhile, social emergency organizations that play a key role in emergency rescue operations should be commended and rewarded. We should form a positive public opinion orientation for emergency responses and create a strong atmosphere for participating in emergency rescues, to encourage and guide social emergency organizations to actively participate in emergency rescue operations in an orderly manner.

## 8. Conclusions

In view of the limitations and problems in data acquisition, result accuracy, and conclusion verifications of previous studies on emergency rescues, this study constructed an evolutionary game model of emergency rescue cooperation in order to further describe the dynamic game relationship between various emergency organizations in rescue operations. This study revealed the formation mechanism and path of evolutionarily stable strategies of the government rescue team, the social emergency organization, and the government support institution. The influence of key parameters on the evolution of emergency strategy combination was studied by simulation analysis. The main conclusions are as follows:

1. The game system of emergency rescue cooperation has a strong integrity, and (1,1,1) or (0,0,0) are the stable strategy combinations of system evolution. The government rescue team is the core subject of the emergency rescue cooperation that evolves toward a stable strategy most quickly in the game system of emergency rescue cooperation. However, the evolution of the social emergency organization and the government support institution toward stable strategies is relatively lagging.
2. The accumulation and mobilization of the emergency capital stock affects the initiative of cooperation and coordination among emergency organizations. The emergency capital stock is an important driving force for the evolution of the emergency rescue cooperation game system toward (1,1,1). Meanwhile, reducing the cooperation redundancy among emergency organizations will avoid the disorderly “pooling” of social emergency organization and improve the efficiency of emergency rescue cooperation.
3. Enhancing the scale and level of cooperation among emergency organizations, strengthening the implementation efficiency of emergency rescue operations, and maintaining the implementation intensity of emergency support measures will promote the formation of an evolutionarily stable strategy (1,1,1).
4. In order to improve the performance of emergency rescue cooperation, emergency resources should be rationally allocated among emergency organizations. At the same time, the improvement of a rescue reward and punishment mechanism and a requisition compensation mechanism will enhance the willingness of emergency organizations to evolve toward positive emergency strategies.

Compared with previous studies, the contribution of this paper is mainly reflected in two aspects. On the one hand, in view of the fact that the existing research rarely considers the cooperative interaction among the government rescue team, the social emergency organization, and the government support institution at the same time, we extend the main body of the model and put the three into the same game system, which increases the systematicness and completeness of emergency rescue cooperation decision-making. On the other hand, compared with previous studies, we also introduced parameters, such as emergency capital stock, implementation efficiency of emergency rescues, effect coefficient of emergency cooperation, intensity coefficient of emergency support during emergency rescue cooperation, etc., which further depicts the relevance of emergency rescue scenarios in detail. This approach makes the conclusions more abundant, and also broadens the perspective and scope of describing the emergency rescue cooperation decision-making model. The conclusions and suggestions have theoretical and practical significance for optimizing the efficiency of emergency rescue cooperation.

## Figures and Tables

**Table 1:** Definition of parameters.

Symbol	Definition	Ranges
*I*	The benefits of disaster loss reduction and social security stability gained by the government rescue team through the implementation of emergency operations.	*I* ≥ 0
*M_S_*	The incentive and compensation income obtained by the government support institution under the positive response strategy.	*M_S_* ≥ 0
*C_G_*	The organization, manpower, equipment, and technical costs paid by the government rescue team to achieve the rescue target and carry out the emergency rescue work.	*C_G_* ≥ 0
*C_E_*	The organization cost and communication transaction cost paid by the social emergency organization participating in emergency rescues.	*C_E_* ≥ 0
*C_S_*	The cost paid by the government support institution to provide various support measures, emergency procurement, compensation, and reward for the rescue organization.	*C_S_* ≥ 0
*G*	The rewards and support obtained by the social emergency organization from government departments.	*G* ≥ 0
*T*	The compensation for the cost of manpower, equipment, and materials paid by the social emergency organization from the governments.	*T* ≥ 0
*L*_1_	Total perception of losses caused by emergencies and derivative disasters for the government rescue team.	*L*_1_ ≥ 0
*L*_2_	Total perception of losses caused by emergencies and derivative disasters for the government support institution.	*L*_2_ ≥ 0
*P_G_*	The punishment and accountability obtained by the government rescue team from the superior governments.	*P_G_* ≥ 0
*P_S_*	The punishment and accountability obtained by the government support institution from the superior governments.	*P_S_* ≥ 0
*R*	The benefits of trust and social reputation obtained by the social emergency organization from the recognition of the government departments.	*R* ≥ 0
*E*	The additional support costs paid by the emergency rescue organizations to maintain the rescue efficiency of the government rescue team and the social emergency organization.	*E* ≥ 0
*A*_1_	The emergency capital stock formed between the government rescue team and the social emergency organization based on trust relationships and institutional norms.	*A*_1_ ≥ 0
*A*_2_	The emergency capital stock formed between the government support institution and the social emergency organization based on information matching.	*A*_2_ ≥ 0
*H_G_*	The additional management costs paid by the government rescue team to dissipate the redundancy of emergency rescue cooperation.	*H_G_* ≥ 0
*H_E_*	The additional management costs paid by the social emergency organization to dissipate the redundancy of emergency rescue cooperation.	*H_E_* ≥ 0
*α*	The implementation efficiency of emergency rescues.	0 ≤ *α* < 1
*θ*	The effect coefficient of emergency cooperation.	0 ≤ *θ* < 1
*ω*	The intensity coefficient of emergency support.	0 ≤ *ω* ≤ 1

**Table 2:** The payoff matrix under the government support institution’s positive response strategy.

				The Government Support Institution
				Positive Response (*z*)
**The government rescue team**	**Positive collaboration (*x*)**	**The social emergency organization**	Orderly participation (*y*)	$\begin{array}{c} I-\left(C_{G}-A_{1}\right)-\left(1-\alpha \right)\left(1-\theta \right)L_{1}-\left(1-\alpha \right)\left(1-\theta \right)H_{G},\\ G+T+R-\left(C_{E}-A_{1}-A_{2}\right)-\left(1-\alpha \right)\left(1-\theta \right)H_{E},\\ M_{S}-\left(C_{S}-A_{2}\right)-\left(1-\alpha \right)L_{2} \end{array}$
			Disordered participation (1 − *y*)	$\begin{array}{c} I-C_{G}-\left(1-\alpha \right)L_{1}-\left(1-\alpha \right)H_{G},\\ T-C_{E},\\ M_{S}-C_{S}-\left(1-\alpha \right)L_{2}-E \end{array}$
	Negative collaboration (1 − *x*)		Orderly participation (*y*)	$\begin{array}{c} I-C_{G}-L_{1}-P_{G},\\ G+T+R-\left(C_{E}-A_{2}\right)-\left(1-\alpha \right)H_{E},\\ M_{S}-\left(C_{S}-A_{2}\right)-L_{2}-E \end{array}$
			Disordered participation (1 − *y*)	$\begin{array}{c} I-C_{G}-L_{1}-P_{G},\\ T-C_{E},\\ M_{S}-C_{S}-L_{2}-E \end{array}$

**Table 3:** The payoff matrix under the government support institution’s negative response strategy.

				The Government Support Institution
				Negative Response (1 − *z*)
The government rescue team	Positive collaboration (*x*)	The social emergency organization	Orderly participation (*y*)	$\begin{array}{c} I-\left(C_{G}-A_{1}\right)-\left(1-\theta \right)L_{1}-\left(1-\theta \right)H_{G},\\ \varpi \left(G+T\right)+R-\left(C_{E}-A_{1}\right)-\left(1-\theta \right)H_{E},\\ -\varpi C_{S}-L_{2}-P_{S} \end{array}$
			Disordered participation (1 − *y*)	$\begin{array}{c} I-C_{G}-L_{1}-H_{G},\\ \varpi T-C_{E},\\ -\varpi C_{S}-L_{2}-P_{S} \end{array}$
	Negative collaboration (1 − *x*)		Orderly participation (*y*)	$\begin{array}{c} I-C_{G}-L_{1}-P_{G},\\ \varpi \left(G+T\right)+R-C_{E}-H_{E},\\ -\varpi C_{S}-L_{2}-P_{S} \end{array}$
			Disordered participation (1 − *y*)	$\begin{array}{c} I-C_{G}-L_{1}-P_{G},\\ \varpi T-C_{E},\\ -\varpi C_{S}-L_{2}-P_{S} \end{array}$,

## Data Availability

Data sharing not applicable.
